# Protein Fractions from Flaxseed: The Effect of Subsequent Extractions on Composition and Antioxidant Capacity

**DOI:** 10.3390/antiox12030675

**Published:** 2023-03-09

**Authors:** Katarzyna Waszkowiak, Beata Mikołajczak, Katarzyna Polanowska, Marek Wieruszewski, Przemysław Siejak, Wojciech Smułek, Maciej Jarzębski

**Affiliations:** 1 Department of Gastronomy Science and Functional Foods, Faculty of Food Science and Nutrition, Poznan University of Life Sciences, Wojska Polskiego 31, 60-624 Poznań, Poland; 2 Department of Meat Technology, Faculty of Food Science and Nutrition, Poznan University of Life Sciences, Wojska Polskiego 31, 60-624 Poznań, Poland; 3 Department of Food Technology of Plant Origin, Faculty of Food Science and Nutrition, Poznan University of Life Sciences, Wojska Polskiego 31, 60-624 Poznań, Poland; 4 Department Mechanical Wood Technology, Faculty of Forestry and Wood Technology, Poznan University of Life Sciences, Wojska Polskiego 28, 60-637 Poznań, Poland; 5 Department of Physics and Biophysics, Faculty of Food Science and Nutrition, Poznan University of Life Sciences, Wojska Polskiego 38/42, 60-637 Poznań, Poland; 6 Institute of Chemical Technology and Engineering, Poznan University of Technology, Berdychowo 4, 60-695 Poznań, Poland

**Keywords:** flaxseed, proteins extraction, antiradical activity, antioxidant capacity, emulsion, phenolic compounds

## Abstract

Flaxseed proteins exhibit functionalities interesting for the food industry, including antioxidant capacity. Antioxidant activity depends on the protein composition and the presence of phenolic compounds extracted with them from the matrix. The research focused on the effect of subsequent protein extractions (water, salt and alkaline) of flaxseed meals (of three cultivars) on the protein fraction composition and its relations to antioxidant capacity. The protein and phenolic profiles and antioxidant functionalities (in antiradical ORAC and emulsion assays) were analysed. Spectroscopic characteristics of the fractions (fluorometric and FT-IR analysis) were also included. Our study has shown the effect of fractionation on the share of proteins at MW from 56–38 kDa (globulin-like) and <15 kDa (albumin-like) in the protein profiles. The highest globulin share was in the alkaline-extracted fractions (AEF) and albumin in the salt-extracted (SEF) ones. SDG (secoisolariciresinol diglucosides) and phenolic acids (*p*-coumaric and ferulic) were extracted with flaxseed proteins. Their contents were fraction-dependent and the highest in AEF. The concentration of phenolics in AEF corresponded with the highest antiradical capacity (ORAC) compared with the other fractions. However, the SEF showed a higher ability to inhibit oxidation in emulsions than AEF, which could be associated with the higher content of the low MW proteins.

## 1. Introduction

Flaxseed (*Linum usitatissimum* L.) is one of the crucial oilseeds [1], which is a source of oil rich in essential n-3 fatty acids (α-linolenic acid, ALA). Flaxseed, as well as flaxseed cakes or meals, products remaining after pressing flaxseed oil, are also a source of various compounds, such as proteins, mucilage and lignans [2]. These compounds can be valuable for food production from both nutritional and functional points of view [3].

After industrial oil extraction, the protein content in defatted flaxseed meals is high and amounts to between approximately 35 and 45% of the dry oil-free mass [4]. The biological value of flaxseed protein is also significant compared to other plant proteins. Their essential amino acid composition and digestibility are comparable to pea protein and soy protein [5,6]. The flaxseed protein-derived peptides (products of their hydrolysis) showed various biological activities, such as antioxidant capacity, angiotensin-converting enzyme inhibition, antibacterial activity, anti-inflammatory and anti-diabetic effects [7]. Moreover, the flaxseed protein’s primary structure, relatively ordered secondary structure, tunable structural properties and suitable interfacial behaviours indicate that their isolates and concentrates can exhibit interesting functional properties for food production [8]. They include foaming and emulsifying properties [6]. All these nutritional and functional benefits encourage researchers to focus on methods of efficient extraction and isolation or concentration of flaxseed proteins [9,10]. In studies by Qin et al. [11] and Nwachukwu et al. [12], the flaxseed proteins were extracted and fractionated to obtain albumin and globulin and then their composition and selected functional properties were compared.

In the flaxseed matrix, the proteins occur in natural associations/complexes with mucilage and phenolic compounds. The compounds can be extracted with the flaxseed proteins, influencing their functional properties, including antioxidant activity. Flaxseed mucilage is a viscous seed coat gum composed of neutral and acidic polysaccharides [13]. The neutral polysaccharides are arabinoxylans consisting of L-arabinose, D-xylose and D-galactose, while the acidic polysaccharides are rhamnogalacturonans containing L-rhamnose, L-fucose, L-galactose and D-galacturonic acid. Flaxseed mucilage shows various interesting functional properties, including water holding and oil binding capacity, emulsifying activity, water–oil emulsion stability and gelling and coating properties [8]. A ratio of neutral to acidic polysaccharides influences the physicochemical properties of flaxseed mucilage, such as viscosity. It may affect the listed functionalities. Because mucilage is a soluble dietary fibre, it also possesses probiotics, anti-obesity, anti-cholesterol and anti-diabetic activities [14,15,16].

The main flaxseed phenolic compounds are lignans—secoisolariciresinol diglucosides (SDG) and phenolic acid glucosides (including ferulic and *p*-coumaric acid derivatives) [17]. In the flaxseed matrix, these phenolic compounds form large complexes (oligomers), which are composed of SDG molecules ester-linked with 3-hydroxy-3-methylglutaryl (HMG) residues and other hydroxycinnamic acid derivatives [18]. SDG has confirmed antioxidant capacity, which is crucial for the health benefits provided by this molecule [19]. Some of the flaxseed phenolic compounds are probably extracted together with mucilage or proteins during the extraction process and may affect the antioxidant properties of the resulting concentrates [20,21].

Although flaxseed contains significant amounts of SDG and phenolic acids, it remains a question whether their extraction together with flaxseed proteins is efficient and how much they participate in the antioxidant activity of the received protein fractions. Recent studies focused on the extraction process and structural modification of flaxseed proteins to improve their functionalities, e.g., emulsifying properties [6]. Some researchers also studied the effect of these processes on the antioxidant activity of flaxseed proteins [11,20] using various analytical assays, e.g., spectrophotometric 2,2-diphenyl-1-picryl-hydrazyl radical (DPPH^•^) or ferric reducing antioxidant power (FRAP) assays. However, there is a lack of information on whether the results correspond with the protein antioxidant capacity in an emulsion.

The article showed the research results on the effect of the subsequent protein extractions (water extraction, salt extraction and alkaline extraction) of flaxseed meals on the protein fraction composition (including spectroscopic characterisation, amino acid, electrophoretic and phenolic compound profiles) and antioxidant functionalities (studied both in analytical ORAC assay and emulsion).

## 2. Materials and Methods

### 2.1. Material and Reagents

The research materials were the protein fractions of flaxseed (*Linum usitatissimum* L.) obtained from of three cultivars: golden-seed Jantarol, Oliwin and brown-seed Szafir (IHAR, Borowo, Poland; year of production: 2017; for the chemical composition of the material see Waszkowiak and Mikołajczak [22]). The material preparation included grinding in a ZM 200 mill (1 mm sieve, Retsch, Haan, Germany), double cold oil extraction (ground seed to n-hexane ratio of 1:3 *w*/*v*) and then grinding in a colloidal mill (Foss, Hilleroed, Denmark) to standardise the material composition. The defatted meals were cold-stored at 4 °C.

Folin–Ciocalteu reagent (FCR), 6-hydroxy-2,5,7,8-tetramethylchromane-2-carboxylic acid (Trolox), 8-anilinonaphthalene-1-sulfonic acid (ASN), butylated hydroxytoluene (BHT) and Tween 20 were from Sigma-Aldrich (Munich, Germany). Analytical standards of SDG were from PhytoLab (Vestenbergsgreuth, Germany) and standards of phenolic acids and enzyme β-glucosidase from almond (2 units mg^−1^ solid) were from Merck (Darmstadt, Germany). Other solvents and reagents (analytical (ACS) or HPLC grade) were purchased from Merck (Darmstadt, Germany), SERVA Electrophoresis (Munich, Germany) or POCH (Gliwice, Poland).

Spectra/Por molecularporus membrane tubing (membrane 1, MWCO:6–8 kDa) were purchased from Spectrum Medical Industries Inc. (Huston, TX, USA).

### 2.2. Extraction Process

**Figure 1:**
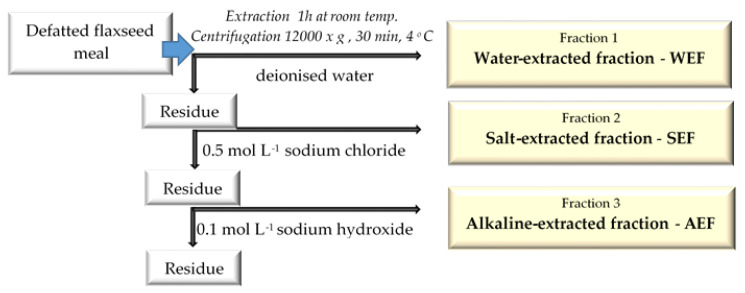
Protocol subsequent extractions of protein fractions from defatted flaxseed meal.

Protein extraction from defatted flaxseed meals and subsequent fractionations were carried out according to the procedure described by Kwon et al. [23] with some modifications. In this procedure, protein extraction and fractionation are based on differences in solubility (Figure 1).

Briefly, defatted meal (20 g) was mixed with deionised water (1:10 and 1:15 *w*/*v* for Szafir/Oliwin and Jantarol cultivar, respectively) and stirred continuously for 1 h at room temperature. The higher meal-to-water ratio for Jantarol seed extraction was due to the higher content of water-soluble mucilage in these seeds, which increased the viscosity of the solution compared to the other cultivars.

Then, the pH of solution was adjusted to 4.2 using 1 mol L^−1^ hydrochloric acid and centrifuged (12,000× *g* for 30 min at 4 °C; Heraeus Megafuge 40R centrifuge, Thermo Fisher Scientific, Santa Clara, CA, USA). The precipitate (residue) was used for further extraction, while the supernatant was collected, filtered using glass wool and dialysed against deionised water using from 6–8 kDa MWCO dialysis membrane tubing for approximately 24 h at 4 °C (with at least six changes of water). The content of the dialysis membrane tubing was freeze-dried (Alpha 1–4 LSC Freeze dryer; Christ, Germany) and labelled as WEF (water-extract fraction).

The residue after first extraction was dissolved in 150 mL of 0.5 mol L^−1^ sodium chloride, and the pH of solution was adjusted to 8.0. The solution was stirred continuously for 1 h at room temperature, and then centrifuged (12,000× *g* for 30 min at 4 °C). The residue was used for further extraction, while the supernatant was collected and dialysed against deionised water using from 6–8 kDa MWCO dialysis membrane tubing for approximately 24 h at 4 °C. The content of the dialysis membrane tubing was freeze-dried and labelled as SEF (salt-extracted fraction).

The residue after the second extraction was dissolved in 150 mL of 0.1 mol L^−1^ sodium hydroxide. The pH of solution was adjusted to 11.0 using 1 mol L^−1^ sodium hydroxide. The next extraction of proteins was performed for 1 h at room temperature with continuous stirring, followed by centrifugation (12,000× *g* for 30 min at 4 °C). The supernatant was collected and dialysed as previously described. The obtained fraction labelled AEF (alkaline-extract fraction) was freeze-dried. All flaxseed protein factions were stored at 4 °C in the dark for further use.

### 2.3. Amino Acid and Electrophoretic Profile Analyses

The amino acid profiles of protein fractions were determined by an high-performance liquid chromatography (HPLC) gradient system with precolumn phenylisothiocyanate (PITC) derivatisation after acid hydrolysis in 6 mol L^−1^ hydrochloric acid with 1% phenol under nitrogen at 110 °C over 24 h, as proposed by Kwanyuen and Burton [24]. The tryptophan content was examined after alkaline hydrolysis of proteins in 4 mol L^−1^ sodium hydroxide at 110 °C over 18 h under nitrogen and analysed according to the method proposed by Çevikkalp et al. [25] with slight modifications. The analysis was performed using the LC Agilent Technologies 1200 Rapid Resolution (Santa Clara, CA, USA) system equipped with a UV-Vis detector DAD 1260 and a reversed-phase column Zorbax Eclipse Plus C18 (4.6 × 150 mm, 5 µm). Two independent samples for each fraction were prepared and each sample was injected twice.

The electrophoretic protein separation (SDS-PAGE) of protein fractions was carried out according to the procedure described by Waszkowiak et al. [26] under reducing conditions. The separation was performed in 15% polyacrylamide separating gel. The PageRuler Plus Protein Ladder from 10–250 kDa (Thermo Fisher Scientific, Waltham, MA, USA) was run as the standard. The protein content in each separated sample was 12 μg (it was measured with a 2-D Quant Kit, GE Healthcare Bio-Sciences, Marlborough, MA, USA). The gels obtained as a result of electrophoretic separation were scanned using a Molecular Imager^®^ Gel Doc^TM^ XR+ System scanner (Bio-Rad Laboratories, Inc. Hercules, CA, USA). Gel path analysis was developed using Image Lab 6.0.1 software (Bio-Rad Laboratories, Inc.). All bands accounted for 100%, the shares of individual protein bands were determined and analysed statistically.

### 2.4. SDG and Phenolic Acid Analysis

The qualitative and quantitative analysis of lignans and main phenolic acids of flaxseed protein fractions was preformed according to the procedure described by Renouard et al. [27] and Fuentealba et al. [28] with some modification. The procedure included alkaline and enzymatic (β-glucosidase) hydrolyses, followed by high-performance liquid chromatography (HPLC).

An amount of 0.100 g of each fraction was weighed and 1.8 mL of deionised water was added. The samples were dissolved by periodically mixing (for 30 min at room temperature by vortex) and then underwent sonication using an ultrasonic bath (10 min at room temperature). The procedure of mixing and sonication was repeated.

A volume of 0.2 mL sodium hydroxide at a concentration of 1 mol L^−1^ was added to the dissolved samples (0.1 mol L^−1^ final concentration). Hydrolysis was carried out at 50 °C for 12 h (Thermoblock TB-951U, JW Electronic, Warszawa, Poland). Then, the sample was cooled down and neutralised by the addition of 2 mol L^−1^ hydrochloric acid. The 0.5 mL of neutral hydrolysate sample was transferred to an Eppendorf test tube and methanol was added (volume ratio 1:1) to precipitate proteins and mucilage. The sample was left for 24 h at 4 °C, and then centrifuged at 10,000× *g* for 10 min (Espresso Personal Microcentrifuge, Thermo Scientific, Waltham, MA, USA). The supernatant was filtered by an RC syringe filter (diameter: 13 mm, pore size: 0.45; Thermo Fisher Scientific, Santa Clara, CA, USA) and submitted to lignan SDG analysis by HPLC.

After alkaline hydrolysis, 0.5 mL of neutralised samples were taken, evaporated to dryness at 38 °C (Genvac miVac Duo Centrifugal Concentrator, Thermo Fisher Scientific), and suspended in 0.5 mL of β-glucosidase solution (2 U mL^−1^ of enzyme at 0.1 mol L^−1^ sodium acetate buffer, pH 5). The samples were hydrolysed for 12 h at 40 °C in the SIF 6000R control temperature oven (Lab Companion, Billerica, MA, USA) under gentle shaking. Methanol was added (volume ratio 1:1) to stop hydrolysis and the samples were cleaned as was described above. The supernatants were submitted to phenolic acid analysis by HPLC.

The main flaxseed phenolic compounds, i.e., secoisolariciresinol diglucosides (SDG), ferulic acid and *p*-coumaric acid, were identified and quantified by the HPLC method using an Agilent 1290 Infinity LC System (Agilent, Santa Clara, CA, USA) equipped with a Luna Omega 5 µm Polar C18 100A LC Column (4.6 × 75 mm; Phenomenex, Torrance, CA, USA). The injection volume was 20.0 µL. A mobile phase gradient with acetonitrile (solvent A) and 1% aqueous solution of acetic acid (solvent B) was developed: linear increment from 12% to 60% of solvent A in 18 min, further increase of A to 70% in 1 min and followed by a decrease to 12% of A in 1 min at a constant flow rate of 0.3 mL∙min^−1^. The eluate was monitored using a Diode-Array detector (DAD) set at the wavelength characteristic for the analysed compounds. The identification and quantification of phenolic compounds was conducted by comparing their retention times with those of corresponding standards; additionally, a DAD detector was applied to identify the compounds on the basis of their absorption spectra. Two independent samples for each fraction were prepared and each sample was injected twice. The results were expressed in mg per gram of fraction.

### 2.5. Spectroscopic Characterisation of the Protein Fractions

#### 2.5.1. Fourier Transform Infrared (FT-IR) Analysis

FT-IR spectra were determined by the spectrum Two FT-IR spectrometer with a Universal ATR with a diamond crystal (PerkinElmer, Waltham, MA, USA). The experiment was set up for all available spectral ranges (500–4000 cm^−1^). Typically, a microgram of the sample was put on the diamond crystal in the ATR system with a controlled force gauge. The measurements were conducted three times for each fraction sample.

#### 2.5.2. Fluorometric Analysis

The fluorescence spectra (emission and excitation) of each fraction sample (solution in double-deionised water at a concentration of 0.1 g L^−1^) were recorded by the Shimadzu RF 5001PC fluorometer (Kyoto, Japan) at ambient conditions. All the spectroscopic measurements were conducted at ambient conditions in a 1 cm × 1 cm quartz cuvette. Samples were excited at the UV and VIS region (200–500 nm), and emission was measured over the range up to 990 nm with 3 nm excitation and emission slits. Geometry excitation to the emission beam was 90° (L-shaped).

### 2.6. Functional Properties of Flaxseed Protein Fractions

Stock solutions of the protein fractions in deionised water (10 g L^−1^) were prepared by periodical mixing in a vortex. Next, they were centrifuged at 15,000× *g* for 15 min at 4 °C (Heraeus Megafuge 40R centrifuge) and used for the further determination of protein surface hydrophobicity, total phenolic content and antioxidant activity by ORAC_FL assay and in emulsion. Two independent samples for each fraction were prepared and each sample was analysed twice.

#### 2.6.1. Protein Surface Hydrophobicity (H_o_)

Protein surface hydrophobicity was analysed by the method using anionic (8-anilinonaphthalene-1-sulfonic acid, ASN) fluorescent probes [29].

The stock solutions of the protein fractions were serially diluted to final concentrations ranging from 0.0063–0.1 g L^−1^ (*w*/*v*) in 0.01 mol L^−1^ sodium phosphate buffer (pH 7.0). A 15 µL aliquot of ANS solution (11 mg mL^−1^) was added to 3 mL of serially diluted sample solutions, mixed thoroughly, and the fluorescence intensity was measured using a florescence spectrophotometer (Hitachi F-2700, Hitachi High Technologies, Pleasanton, CA, USA; 390 nm excitation and 490 nm emission wavelength, 5 nm widths of excitation and emission slit).

The results were expressed as the index of protein surface hydrophobicity H_o_, which is defined as the initial slope of the plot of the relative fluorescence intensity (RFI) vs. protein concentration, calculated by the simple linear regression. The relative fluorescence intensity was counted as follows [30]: RFI = (F − F_0_)/F_0_, where F is the fluorescence of the protein–ANS conjugate, and F_0_ is the fluorescence of the ANS solution without protein fractions.

#### 2.6.2. Antiradical Activity (ORAC_FL Assay)

The oxygen radical absorbance capacity assay with fluorescein sodium salt (Fluka, Everet, WA, USA) as a fluorescent probe (ORAC_FL) by Ou et al. [31] was carried out following the previously described procedure [32]. The fluorescence was recorded using a florescence spectrophotometer (Hitachi F-2700; 493 nm excitation and 515 nm emission wavelength).

The stock solutions were diluted with deionised water to obtain appropriate concentrations within the assay activity range. Trolox solutions (0–100 μmol L^−1^) were used as standards. The results were calculated by subtracting the areas under the fluorescein decay curves for the control sample (without standard or protein fraction) and the test sample (net area) and were expressed as μmol of Trolox equivalents (TE) per gram of the sample.

#### 2.6.3. Total Phenol Content (TPC)

The total phenol content was determined by the Folin–Ciocalteu method [33,34]. The method is based on the spectrophotometric measurement of the intensity of the colorimetric reaction that takes place between the phenolic groups (derived from the standard or sample) with the Folin–Ciocalteu reagent (FCR) in the sodium carbonate environment.

The sample (0.040 mL of standards, 0.100 mL of WEF and 0.080 mL of SEF and AEF, respectively) was mixed with deionised water up to a total volume of 3.200 mL. As a blank, 3.200 mL µL of deionised water was used. Then, 0.200 mL of Folin–Ciocalteu reagent was mixed with the sample and, after 5 min, 0.200 mL of aqueous sodium carbonate solution (200 g L^−1^) was added. After 120 min of incubation in the dark at room temperature, the absorbance was measured at 765 nm against a blank. The calibration curve of gallic acid (the standard solution: 0–0.9 mg mL^−1^) was used to calculate the total phenol contents and the results were expressed as gallic acid equivalents (GAE) in milligrams per gram of the sample.

#### 2.6.4. Ability to Inhibit Oxidation in Emulsion System

The test emulsion containing 2.5% flax oil (received from a local manufacturer) and 0.25% Tween 20 in 0.05 mol L^−1^ potassium phosphate buffer (pH 7.2) was prepared as described by Waszkowiak and Barthet [35]. Fresh emulsion was prepared on the day the oxidation test was performed. An aliquot (5 mL) of the emulsion was transferred into 25 × 95 mm glass screw cap tubes. Then, 25 µL of additive was added and the emulsion mixture was vortexed thoroughly. The additives were (per amount of oil in emulsion) the known antioxidant butylated hydroxytoluene (BHT; 0.1 g L^−1^) and three protein fractions (10 g L^−1^). A control emulsion without additive was run with each oxidation study.

The oxidation test was performed over 24 h at 40 °C in the dark in an incubator (SIR6000R, Lab Companion, Billerica, MA, USA) with constant mixing (110 random per min). In fresh emulsions (initial value), and after a 24 h-oxidation test, the extractions of oil were performed by adding 2.5 mL of hexane and 2.5 mL of isopropanol to 5 mL of emulsion mixture. The sample was thoroughly shaken for 5 min, and then centrifuged for 10 min at 5500× *g* (Heraeus Megafuge 40R centrifuge). The top layer, which contained the oil was removed, diluted with hexane to an appropriate concentration and analysed for conjugated dienoic derivatives (CD) and trienoic derivatives (CT) according to the AOCS Official Method Th 1a-64 [36] and Pegg’s protocol [37], respectively

Two sample tubes of each emulsion (test and control) were analysed to monitor lipid oxidation and two independent analyses were performed for each sample.

The ability of the selected additives to inhibit oxidation in the emulsion system was expressed as PI_24 h (the protective index after 24-h incubation of emulsion at 40 °C). The index was defined as the CD/CT value of the sample with the additive divided by the CD/CT value of the control sample. PI < 1 denotes an acceleration effect on the oxidation in emulsion, PI = 1 denotes no effect on oxidation, and PI > 1 denotes a protective effect against oxidation [38].

### 2.7. Statistical Analysis

The experiments were performed for two independently prepared fraction samples. Each analysis was determined at least in duplicate. All experimental results were expressed as mean ± standard deviation. Statistical analyses were conducted using Statistica software (v.13.3, Tibco Software Inc., Palo Alto, CA, USA). The effects of subsequent extractions of protein fractions (L = 3; water-extraction—WEF, salt extraction—SEF and alkaline-extraction—AEF) and flax cultivars (L = 3; Szafir, Oliwin, Jantarol) on dependent variables were analysed. Analysis of variance (ANOVA) for a completely randomised design (CRD) experiment was carried out, and post hoc Tukey’s HSD test was applied for multiple comparisons of the means (at a significance level of α = 0.05). A Pearson correlation coefficient (*r*) was computed to assess the linear relationship between variables. In the statistical analysis of the results, *p*-values < 0.05 were taken as statistically significant.

## 3. Results and Discussion

### 3.1. Electrophoretic and Amino Acid of Profiles of the Flaxseed Protein Fractions

**Figure 2:**
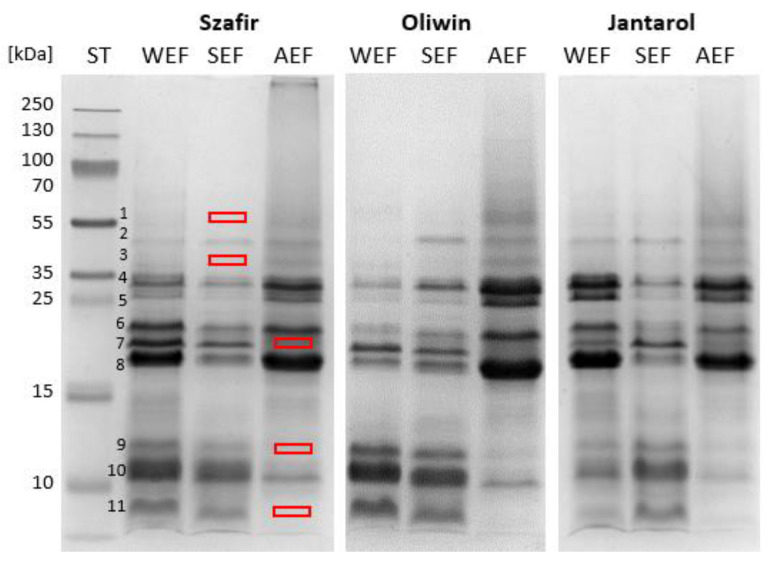
SDS-PAGE profiles of the flaxseed protein fractions. ST—standard, molecular weight [kDa]; WEF—water-extracted fraction; SEF—salt-extracted fraction; AEF—alkaline-extracted fraction; Szafir, Oliwin and Jantarol cultivars. The rectangles indicate the disappearing bands 1, 3 for SEF and 7, 9, 11 for AEF fractions, respectively.

The results confirm that the subsequent extractions and fractionation affects protein contents, amino acid compositions and SDS-PAGE electrophoretic profiles of the flaxseed protein fractions (Table 1 and Figure 2, respectively).

The total protein content (based on the sum of amino acids, Table 1) was the highest in AEF (alkaline-extracted fractions) compared to WEF (water-extracted) and SEF (salt-extracted) fractions, irrespective of the flax cultivar. It ranged from 500.4 mg g^−1^–708.1 mg g^−1^ for AEF, 369.7–505.8 mg g^−1^ for SEF (the lowest for Jantarol fractions in both cases), and 311.2–332.5 mg g^−1^ for WEF. The results showed that water extraction and subsequent salt extraction probably removed the mucilage resulting in protein concentration in alkaline-extracted fractions. However, its removal was not total since the protein concentration in AEF fractions did not exceed between 50 and 70%.

The lowest protein concentration in AEF of Jantarol probably resulted from differences in the chemical composition of the selected flaxseed. Our previous study showed [19] that the seeds of Jantarol were characterised by a lower protein content (170.4 g kg^−1^) than the seeds of Szafir and Oliwin cultivars (220.9 kg^−1^ and 213.7 kg^−1^, respectively). Moreover, the Jantarol seeds had a higher amount of soluble fibre (mucilage, 221.0 g kg^−1^) when compared with the other cultivars used in the study (about 209.4 g kg^−1^ and 212.8 g kg^−1^ for Szafir and Oliwin, respectively). The high mucilage content, forming a viscous solution, may have influenced the extraction process from Jantarol seeds.

Eleven protein bands were found and marked in the SDS-PAGE electrophoretic profiles of flaxseed protein fractions (Figure 2). In our study, the globulin monomers (11S) were represented by band 2, and subunits α (35–22 kDa) and β (~18 kDa) were bands 4–6 and 8, respectively. Proteins of the albumin-like group (bands 9–11) were characterised by a molecular weight (MW) <15 kDa. The protein profile and molecular weight distribution are similar to the data presented in previous reports regarding the albumin and globulin fractions [11,12].

The electrophoretic profiles of AEF differed from WEF and SEF, irrespective of the cultivar. In the AEF samples, the lowest percentage share of those albumin-like proteins at low MW (<15 kDa) was observed (8.10%; see Supplementary Materials, Table S1), and proteins at MW ranging from 35 kDa to 16 kDa constituted the largest share (85.79%). It is worth emphasising that bands 7 (~20 kDa), 9 and 11 were not present in the AEF electrophoretic profiles. Fractions WEF and SEF compared to AEF were distinguished by the absence of two bands (1 and 3) at the MW range from 56–38 kDa. WEF and SEF fractions showed similar SDS-PAGE protein profiles. However, the fractions differed in the proportion of particular protein bands. The significantly increased intensities of bands 5 and 8 were in the WEF profile in comparison to SEF.

The statistical analysis of variance (two-way ANOVA, simply main effect) confirmed the significant effect (*p* < 0.001) of subsequent extractions on the share of proteins at MW from 56–38 kDa (globulin-like proteins) and <15 kDa (albumin-like proteins) in the protein electrophoretic profiles. When the fraction type was adopted as a factor, the following dependencies were found for the proteins at MW from 56–38 kDa: WEF < SEF < AEF and for the proteins at MW < 15 kDa: AEF < WEF < SEF, respectively.

When comparing the amino acid profiles, the differences among the fractions were also observed (Table 1). The crucial differences were for the AEF profiles compared to WEF and SEF. AEF fractions were characterised by the highest content of branched-chain amino acids (BCAA; isoleucine and phenylalanine). However, the low amounts of cysteine (sulphur-containing amino acid, SCAA) were in AEF; its percentage share amounted to between 1.6 and 1.7% in the amino acid profiles of AEF. The explanation is the removal of soluble and easy-extractable albumin-like proteins at low MW during water and salt extractions (as shown in the electrophoretic profiles; Figure 2). As it has been proved in previous studies [12,39], the proteins are rich in cysteine. The AA profiles of SEF were characterised by the lower percentage share of hydrophobic amino acids (HAA) in comparison to the AEF profiles, such as alanine, proline and valine, and the highest percentage share of negatively charged amino acids (NCAA).

Our study showed that the subsequent protein extractions allowed to obtain flaxseed protein fractions differed in shares of albumin-like and globulin-like proteins in SDS-PAGE profiles and, therefore, differed in contents of BCAA, SCAA, HAA and NCAA. These differences in the protein fractions resulting from subsequent extractions can influence both their nutritional values and functional properties. The SCAA, HAA and NCAA relate to disulfide bonds, hydrophobic contact, the charged patch on a surface and electrostatic interactions [40,41], which affect protein structural properties and behaviour at the oil–water or air–water interfaces (i.e., emulsifying or foaming properties). The presence of flaxseed proteins at low MW (<15 kDa) in the WEF and SEF fractions and the domination of proteins at higher MW (36–16 kDa) in the AEF fractions can influence those behaviours.

### 3.2. SDG and Phenolic Acid Contents of the Flaxseed Protein Fractions

In flaxseed, most phenolic compounds have a form of glucoside derivatives [42,43]. They form a macromolecular complex in which SDG is ester-linked to HMG; the phenolic acid glucosides (*p*-coumaric acid and ferulic acid derivatives) are linked to this SDG-HMG oligomer [44]. In our study, two-step hydrolysis was applied, which included alkaline hydrolysis followed by enzymatic hydrolysis (with β-glucosidase). Alkaline hydrolysis allows the release of SDG and phenolic acid glucosides from the complex, and enzymatic hydrolysis helped convert phenolic acid derivatives into identifiable free forms [27,28]. The contents of the main flaxseed phenolic compounds in the flaxseed protein fractions, i.e., SDG, ferulic acid and p-coumaric acid, are shown in Table 2. 

The statistical analysis of variance showed the significant effects of fraction type (*p* < 0.001) and cultivar (*p* < 0.001) on the compound contents in the flaxseed fractions. The highest concentrations of SDG and phenolic acids were in the AEF, and the lowest contents were in WEF, regardless of the flaxseed cultivar used as the extraction material. When the variety was adopted as a factor, the following dependencies were found for SDG: Oliwin < Szafir < Jantarol, and for phenolic acids: Oliwin = Szafir < Jantarol, respectively.

The presence of phenolic compounds (lignans and phenolic acids) reported in our study could significantly influence the functional properties of the extracted protein fractions. Pham and co-authors’ study [45] proved that the complexation of flaxseed protein with phenolic compounds gives extra benefits to proteins and protein-based emulsions. They carried out the covalent modification of the flaxseed protein isolate by phenolic compounds (i.e., flaxseed polyphenols (FPP), ferulic acid and hydroxytyrosol (HT)) and the results showed that the surface hydrophobicity could be modified (increase or decrease) by adding selected phenolic compounds. The authors also discovered that the flaxseed protein isolate–FPP-based emulsion had higher oxidative stability than the isolate-based emulsion.

The accumulation of phenolic compounds during extraction, particularly lignan–SDG, could be valuable for the potential antioxidant properties of the flaxseed protein fractions. Our study showed that the highest content of SDG in the protein fractions was after alkaline extraction (AEF). SDG and its aglycone and oligomers extracted from flaxseed shows antioxidant capacity [19,45]. As previously reported, SDG acts, for example, as a radical scavenger and lipid peroxidation inhibitor [18,46,47,48], and its antiradical activities are attributed to the 4-hydroxy-3-methoxy phenyl moiety [47]. The antioxidant capacity of the flax lignan is one of the crucial elements of its beneficial effect on human health [19]. SDG presence in concentrates of flaxseed proteins can enhance their functionalities associated with oxidation inhibition in food.

### 3.3. Spectroscopic Characterisation of the Flaxseed Protein Fractions

#### 3.3.1. FT-IR Spectra Analysis

**Figure 3:**
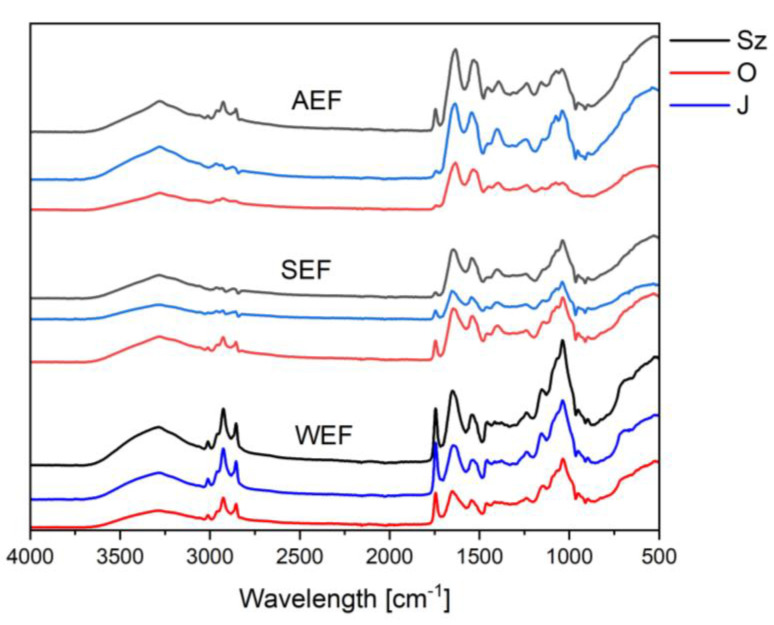
FT-IR spectra of the flaxseed protein fractions. WEF—water-extracted fraction; SEF—salt-extracted fraction; AEF—alkaline-extracted fraction; Szafir (Sz), Oliwin (O), and Jantarol (J) cultivars.

Further information about individual protein fractions provided infrared spectra (Figure 3). Among the signals common to all groups of samples were those originating from stretching vibrations in -OH, =NH and -NH_2_ groups (between 3000 and 3500 cm^−1^), additionally broadened due to the presence of hydrogen bonds. In addition, signals from analogous vibrations of carbon–hydrogen bonds in aliphatic chains (2800–3000 cm^−1^) and the CO bond present in the bonds of amide and ester functional groups (between 1600 and 1750 cm^−1^) were visible. In addition, region between 1500 and 1650 cm^−1^ was characteristic of NH deformation vibrations. At wave number values above 1000 cm^−1^, an intense signal from carbon–oxygen bond vibrations was evident [49]. 

Regardless of the source, the spectra of WEF (the water-extracted fractions) showed a much more intense signal from CH bonds than SEF (salt-extracted) and AEF (alkaline-extracted). Similarly, there is a signal from the carbonyl group at about 1700 cm^−1^, which may indicate a relatively higher amount of ester groups and free carboxylic groups. It could be related to better solubility compounds with a free carboxylic group in the water, which caused them to be in WEF. Moreover, the spectra of WEF showed a more intense sequence of signals in a region between 1200 and 750 cm^−1^ compared with the other fractions (followed by SEF and the lowest in AEF), characteristic of flaxseed mucilage [49,50].

The spectra for all WEF samples were very similar, and the differentiating features were much more pronounced in the other two fractions. In the group of the SEF samples, the relatively high intensity of signals from CH bonds were characterised by the sample from the Oliwin seeds and, among AEF, the fractions extracted from the Szafir cultivar. Since these differences do not relate directly with the data in Table 1, the assumption can stay that they are due to the presence of associated substances extracted with the proteins [51].

#### 3.3.2. Fluorimetric Characterisation

**Figure 4:**
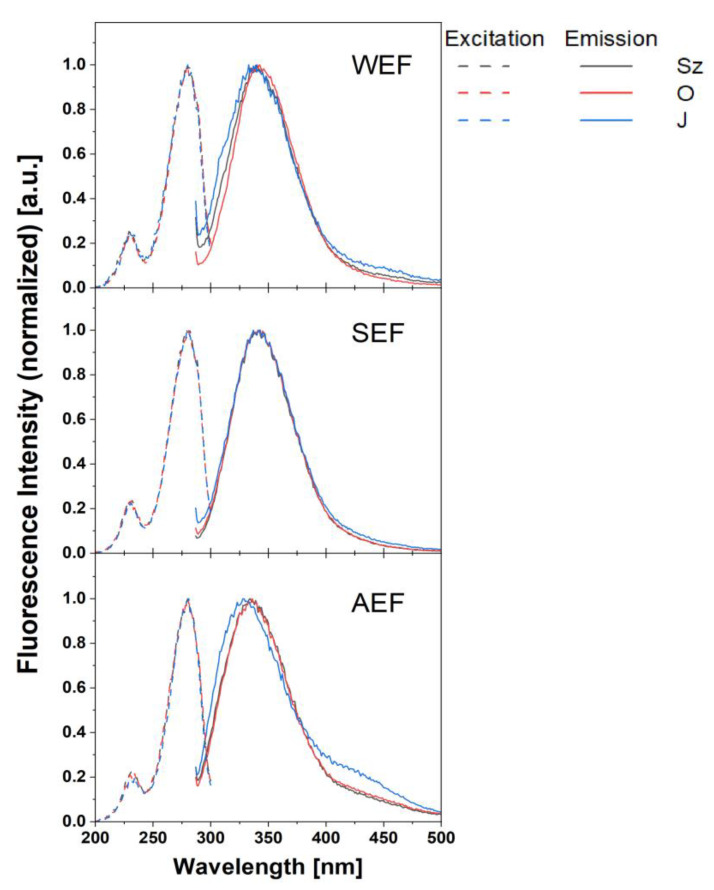
Fluorescence spectra of the flaxseed protein fractions. WEF—water-extracted fraction; SEF—salt-extracted fraction; AEF—alkaline-extracted fraction; Szafir (Sz), Oliwin (O) and Jantarol (J) cultivars; solid lines: emission, dot: excitation.

For further investigations, we have chosen fluorometric characterisation of the protein fractions to determine significant changes originating from eventual impurities or specific inner- or/and intermolecular interactions of compounds. Therefore, several sets of fluorescence emission spectra at different excitation wavelengths (especially UV region: 200–300 nm) were recorded with the step of 10 nm. Additionally, fluorescence excitation spectra were also collected. It was found that the shape of fluorescence emission spectra remained unchanged for individual samples, regardless of the excitation wavelength in the range of excitation from 200–300 nm. For other excitation wavelengths, no emission spectra were recorded. Therefore, the normalised fluorescence excitation (dashed lines) and emission spectra (solid lines) are presented in Figure 4.

Emission spectra of WEF and SEF fractions were characterised by a broad band centred at 340 nm with no regard to the cultivar. However, the blue shift of the fluorescence band was for AEF (band centre from 328–335 nm, cultivar dependent). Moreover, this blue shift was noticeably more pronounced for the Jantarol cultivar (band peak at 328 nm), accompanied by an additional fluorescence band placed at 430 nm, manifested as a shoulder of the main fluorescence band. Similar results to the described, but much less pronounced, were also observed for the WEF of Jantarol. The recorded emission arises mainly from tryptophan with much less contribution of phenylalanine and tyrosine (only those three proteins are fluorescent among all the proteins present in samples). The changes in the spectra can provide information on the molecular organisation of peptides and their interactions within the samples.

The results of the fluorescence excitation spectra allow us to conclude that all recorded emissions come from the same group of chromophores/fluorophores because the fluorescence excitation spectra show the same shape for each sample, regardless of the extraction method, cultivar and wavelength of observation. The observed changes in fluorescence emissions are evidence of the influence of the extraction method on the electronic structure of sample molecules and/or the possibility of the occurrence of intermolecular interactions of compounds. It may occur due to the other compounds’ presence extracted with the proteins, which supports the conclusion drawn from the FT-IR spectra analysis, or conformational changes of proteins due to the extraction conditions [52]. The observed blue shift of fluorescence maximum for AEF and WEF Jantarol cultivars can also suggest the shielding of tryptophan from the environment capable of forming hydrogen bonds. It is the evidence of the different conformation of proteins for the WEF and AEF Jantarol cultivar from proteins in other samples. Among all tested fractions, the AEF from the Jantarol cultivar is the most susceptible to conformational changes. Since observed effects are the most efficient at a high pH, the Jantarol cultivar is the most susceptible to the extraction environment (especially at a higher pH).

### 3.4. Functional Properties of the Flaxseed Protein Fractions

Table 3 shows the results of the analysis of selected protein fractions’ properties, i.e., protein surface hydrophobicity, antiradical activity and total phenolic content.

*Protein surface hydrophobicity (H_o_)*: Higher surface hydrophobicity H_o_ shows that more hydrophobic patches are outside protein molecules. Its value is valuable for studying protein aggregation behaviours and interfacial properties [6].

The results showed that the lowest H_o_ had WEF from the Oliwin and Szafir cultivar, and the highest had AEF from seeds of the Jantarol cultivar. The H_o_ values for the WEF fractions were twice as low as the SEF fractions and between 8 and 9 times lower than AEF, irrespective of the flax cultivar. The H_o_ values of AEF (50.8–80.9) were similar to those reported in previous studies for flaxseed protein concentrates (43.3–68.5; [10]), but lower than those shown for flaxseed protein isolates (120.6; [53]). The study by Nwachukwu and Aluko [12] showed that flaxseed globulins had higher H_o_ than albumins.

The results are consistent with the amino acid composition of the fractions—the significantly higher H_o_ values were for AEF, which were characterised by the higher percentage shear of HAA and lower NCAA in the AA profile compared to the others (Table 1). The statistical analysis (Pearson correlation test) confirmed that protein surface hydrophobicity of the flaxseed protein fractions obtained with subsequent extractions was significantly positively correlated with HAA contents (Pearson correlation coefficient *r* = 0.754, *p* = 0.019).

*Antiradical activity (ORAC_FL)*: ORAC_FL assay measures antioxidant scavenging activity against peroxyl radicals. The analysis (Table 3) showed the lowest activity of WEF fractions. For fractions of the particular flax cultivar, the antiradical activity of SEF was from 1.9 to 3.3 times, and AEF was from 4.8 to 14 times as high as WEF (the lowest for those from the Oliwin cultivar and the highest for the Jantarol fractions, respectively). The results of statistical analysis revealed a strong relationship (correlation coefficient *r* = 0.963, *p* < 0.001) between the antiradical activity of the flaxseed protein fractions and the SDG content (main phenolic compounds, Table 2).

*Total phenolic content (TPC)/compounds reducing Folin–Ciocalteu reagent (FCR)*: In our study, the positive correlation was found between the TPC values and the results of ORAC_FL assay (*r* = 0.974, *p* < 0.001). As for antiradical activity results, the TPC of WEF were the lowest (Table 3). The TPC values of SEF were from 1.4–3 times as high as WEF, and the TPC of AEF were from 2.7–6 times higher than WEF. In the Folin-Ciocalteu method, a phosphotungstate–phosphomolybdate complex is reduced by phenols to blue reaction products measured at 765 nm. Under proper conditions, the assay gives predictable reactions with various phenolics in plants. However, the FCR is significantly reactive towards other nonphenolic compounds, e.g., amino acids (cysteine, tryptophan, tyrosine) and proteins [54]. Therefore, the results for protein fractions reported in our study may have resulted from the reduction of FCR by both phenolic compounds and proteins. This assumption confirmed the results of the statistical analysis, which showed the positive correlation between the TPC values for the protein fractions and SDG contents (Pearson correlation coefficient *r* = 0.938, *p* < 0.001), as well as between the values and hydrophobic amino acid (HAA) contents (*r* = 0.722, *p* = 0.028).

The statistical analysis of variance showed statistically significant (*p* < 0.001) effects of the subsequent extraction (water/salt/alkaline), as well as the cultivars on the above properties. In the analyses performed for each of the studied functional properties (two-way ANOVA, simply main effect), adopting the fraction type and cultivar as a factor, respectively, the following dependencies were: WEF < SEF < AEF and Oliwin < Szafir < Jantarol. 

### 3.5. Ability of Flaxseed Protein Fractions to Inhibit Oxidation in Emulsion

Due to the growing interest of researchers and the food industry in the application of flaxseed protein as emulsifiers [6,8,55], we investigated the ability of the flaxseed protein fractions to inhibit oxidation in the emulsion system (protective effect). CD and CT contents were monitored in oil extracted from the emulsion after 24 h of incubation at 40 °C. Changes in CD content (measured at a wavelength of 234 nm) were related to changes in primary oxidation products and changes in CT (measured at 268 nm) to changes in secondary oxidation products during flaxseed oil oxidation [56].

All flaxseed protein fractions showed a protective effect against oil oxidation in the tested emulsion system (Table 4). After 24 h of incubation, the values of CD and CT indexes for the emulsions with the addition of 10 g L^−1^ flaxseed fraction (per oil in emulsion) were significantly lower than the control emulsion (the protective indexes PI_24 h > 1).

The WEF and AEF were characterised by a similar protective effect against oil oxidation in the emulsion. The effects were also close to the BHT (at concentration of 0.1 g L^−1^). The SEF had a statistically significantly higher protective effect than the other additives, particularly toward CD formation—the CD_PI_24 h values for SEF were 1.5 times as high as the others, irrespective of the flax cultivar. The PI_24 h values counted based on changes in CT were also slightly higher for SEF.

Contrary to antiradical activity (ORAC_FL assay results described in the above section), there was no statistically significant relationship between the ability to inhibit oxidation in emulsion (CD or CT_PI_24 h) and the phenolic compound (SDG) content in flaxseed protein fractions (Pearson correlation coefficients: *r* = −0.152, *p* = 0.696 and *r =* −0.064, *p* = 0.870, respectively). There was also no statistically significant relationship between CD or CT_PI_24 h and TPC (*r* = −0.165, *p* = 0.671 and *r* = −0.188, *p* = 0.628, respectively).

The analysis of variance confirmed a statistically significant (*p* < 0.001) effect of the subsequent extractions of protein fractions (fraction type) and the extraction material (cultivar) on the ability to inhibit oxidation in emulsion (CD/CT_PI_24 h index). However, the order was different compared to antiradical activity (ORAC) results. When the fraction type effect was adopted, the following dependencies found for CD_PI_24 h were AEF = WEF < SEF and for CT_PI_24 h were AEF < WEF < SEF.

Our study showed different behaviour of the flaxseed protein fractions in antiradical assay and emulsion, with AEF showing the best antiradical activity in ORAC_FL and SEF having the best ability to inhibit oxidation in the emulsion system.

The better protective effect against oxidation of the salt-extracted protein fractions (SEF) over the alkaline-extracted fractions (AEF) in the emulsion can be a result of their composition. SEF was characterised by lower protein concentration (Table 1). Moreover, the relative differences in the intensities of infrared signals characteristic for chemical bonds present in mucilage components may suggest its higher content in SEF than in AEF. Flaxseed mucilage is a good emulsifier [57]. Previous studies [58,59] showed that protein-to-mucilage proportion in the flaxseed concentrates influenced their functional properties, including water-binding and emulsifying capacities, and the high-mucilage protein concentrates had better properties than the low-mucilage protein isolates.

Moreover, the SDS-PAGE profiles showed the highest concentration of proteins at low MW (Figure 2) in SEF and the lowest share of such proteins in the AEF profile. They are probably an albumin-like (1,6-2S) conlinin, which together with globulin-like (11-12S) linin, are the main groups of flaxseed proteins. Water-soluble conlinin has a higher content of SCAA and NCAA but lower HAA content than flaxseed globulin [12], providing better emulsion-forming ability. The study by Liu and co-authors [60] proved that conlinin is the major protein associated with flaxseed gum and plays a crucial role in its emulsifying properties (conlinin hydrolysis decreased the gum emulsifying activity and emulsion stability). The higher content of SCAA and NCAA (enhanced reducing property) also suggests a better capacity of albumin-like conlinin to protect against lipid oxidation in a food than globulin-like linin.

Both mentioned compounds (mucilage and low MW proteins) can be crucial to enhance the observed protective effect of SEF against oxidation in the emulsion system compared to AEF. They probably allow for improvement in the oil-in-water emulsion stability, preventing the aggregation of oil droplets, as well as helping to locate the compounds with potential antioxidant activity at the oil–water interface. Further research is necessary to investigate this problem.

The food industry is increasingly interested in flaxseed protein applications, among others, due to their functional (e.g., emulsifying) properties. The presence of phenolic compounds in flaxseed protein fractions, especially SDG with proven antioxidant capacity, indicates another crucial functionality related to lipid oxidation inhibition.

Previous studies have mainly focused on producing only one protein concentrate/isolate in one process from a given batch of seeds/meal [9,10]. Few studies have applied the procedure of subsequent extractions and fractionation of flaxseed proteins [11]. In our research, we use the subsequent extractions (first with water, then salt and alkaline solutions) of the same raw material to investigate and understand the relationship between the fraction composition (protein and phenolic profiles) and antioxidant capacity. However, this approach to the extraction process allows for the full use of one raw material batch for the production of protein concentrates with different functional properties compared to separate ones (e.g., three separate extractions from three flaxseed batches). It is in line with the current trend of food and food additive manufacturing.

Our study shows the influence of the fractionation method on the composition and antioxidant capacity of protein fractions. The knowledge can help to design the process to obtain flaxseed protein concentrates with different potential applications in food. The high concentration of SDG (with proven antiradical potential) and globulin-like proteins at high MW (with a high content of HAA and surface hydrophobicity) in an alkaline-extracted fraction suggest its application in food with high-fat content. The salt-extracted fractions can be better for emulsion-like food. The practical application of this approach needs further study.

## 4. Conclusions

The research provides novel information about the influence of protein fraction composition on antioxidant activity and its relation to extraction solvent. Our study has proved there is a significantly higher content of SDG in alkaline-extracted fractions (AEF), which is related to their higher antiradical capacity (ORAC assay) compared with the others (WEF and SEF). However, the AEF showed a lower ability to inhibit oxidation in the emulsion assay than the salt-extracted fraction (SEF). The SDS-Page separation showed that SEF characterised the higher percentage share of the flaxseed proteins at MW < 15 kDa in the protein profile than AEF. It suggests the crucial role of low-MW proteins in protection against oxidation in oil-in-water emulsions.

## Figures and Tables

**Table 1:** Amino acid composition (mg g^−1^) of the flaxseed protein fractions.

Amino Acids	Szafir Cultivar						Oliwin Cultivar						Jantarol Cultivar					
	WEF	%	SEF	%	AEF	%	WEF	%	SEF	%	AEF	%	WEF	%	SEF	%	AEF	%
Asx	46.65 ± 0.57 ^b^	14	72.78 ± 1.16 ^c^	14.7	112.86 ± 0.49 ^e^	17.8	28.41 ± 0.23 ^a^	9.1	69.33 ± 0.69 ^c^	13.7	122.66 ± 4.52 ^f^	17.3	47.65 ± 0.4 ^b^	15.3	52.73 ± 0.41 ^b^	14.3	85.79 ± 0.24 ^d^	17.1
Glx	76.24 ± 0.96 ^b^	22.9	127.33 ± 1.34 ^e^	25.6	124.72 ± 0.58 ^e^	19.7	89.28 ± 0.88 ^c^	28.6	132.07 ± 0.54 ^f^	26.1	135.27 ± 2.41 ^f^	19.1	62.73 ± 0.20 ^a^	20.2	94.10 ± 0.23 ^d^	25.5	97.28 ± 0.60 ^d^	19.4
Ser	16.11 ± 0.14 ^b^	4.8	22.88 ± 0.07 ^c^	4.6	29.88 ± 0.22 ^e^	4.7	14.64 ± 0.22 ^a^	4.7	24.21 ± 0.11 ^d^	4.8	35.09 ± 0.76 ^f^	5	16.66 ± 0.20 ^b^	5.4	17.05 ± 0.21 ^b^	4.6	24.86 ± 0.29 ^d^	5
Gly	21.39 ± 0.06 ^b^	6.4	31.48 ± 0.18 ^f^	6.3	28.88 ± 0.08 ^e^	4.6	25.93 ± 0.03 ^d^	8.3	34.12 ± 0.07 ^h^	6.7	32.64 ± 0.47 ^g^	4.6	17.38 ± 0.06 ^a^	5.6	26.08 ± 0.15 ^d^	7.1	24.37 ± 0.29 ^c^	4.9
His	6.29 ± 0.35 ^b^	1.9	9.04 ± 0.07 ^d^	1.8	12.54 ± 0.03 ^f^	2	4.89 ± 0.16 ^a^	1.6	8.04 ± 0.10 ^c^	1.6	14.54 ± 0.12 ^g^	2.1	7.57 ± 0.06 ^c^	2.4	6.51 ± 0.04 ^b^	1.8	10.98 ± 0.45 ^e^	2.2
Arg	27.86 ± 0.11 ^b^	8.4	42.73 ± 0.61 ^d^	8.6	54.15 ± 0.10 ^e^	8.6	26.74 ± 0.11 ^b^	8.6	42.39 ± 0.04 ^d^	8.4	62.40 ± 0.63 ^f^	8.8	25.09 ± 0.56 ^a^	8.1	29.77 ± 0.23 ^c^	8.1	43.12 ± 0.59 ^d^	8.6
Thr	12.86 ± 0.51 ^b^	3.9	16.00 ± 0.01 ^c^	3.2	23.03 ± 0.22 ^e^	3.6	10.20 ± 0.02 ^a^	3.3	16.90 ± 0.20 ^c^	3.3	26.30 ± 0.60 ^f^	3.7	12.68 ± 0.10 ^a^	4.1	13.44 ± 0.07 ^b^	3.6	19.30 ± 0.02 ^d^	3.9
Ala	13.48 ± 0.18 ^c^	4.1	17.44 ± 0.18 ^d^	3.5	23.34 ± 0.05 ^f^	3.7	11.57 ± 0.20 ^a^	3.7	17.11 ± 0.04 ^d^	3.4	28.65 ± 0.40 ^g^	4	12.47 ± 0.05 ^b^	4	12.99 ± 0.18 ^bc^	3.5	19.35 ± 0.07 ^e^	3.9
Pro	10.98 ± 0.03 ^b^	3.3	13.78 ± 0.32 ^c^	2.8	21.65 ± 0.07 ^e^	3.4	8.32 ± 0.04 ^a^	2.7	14.66 ± 0.02 ^c^	2.9	27.36 ± 0.74 ^f^	3.9	11.44 ± 0.21 ^b^	3.7	10.65 ± 0.05 ^b^	2.9	18.50 ± 0.13 ^d^	3.7
Tyr	8.82 ± 0.06 ^b^	2.7	11.44 ± 0.16 ^c^	2.3	19.11 ± 0.17 ^e^	3	7.39 ± 0.12 ^a^	2.4	11.32 ± 0.19 ^c^	2.2	19.76 ± 0.85 ^e^	2.8	8.21 ± 0.03 ^b^	2.6	8.78 ± 0.07 ^b^	2.4	14.06 ± 0.48 ^d^	2.8
Val	15.14 ± 0.40 ^b^	4.6	19.03 ± 0.07 ^c^	3.8	30.78 ± 0.06 ^e^	4.9	11.84 ± 0.05 ^a^	3.8	19.70 ± 0.04 ^c^	3.9	37.35 ± 1.04 ^f^	5.3	15.23 ± 0.15 ^b^	4.9	14.89 ± 0.26 ^b^	4	26.54 ± 0.48 ^d^	5.3
Met	5.62 ± 0.04 ^a^	1.7	7.18 ± 0.07 ^b^	1.4	10.65 ± 0.39 ^e^	1.7	5.44 ± 0.01 ^a^	1.7	8.10 ± 0.29 ^c^	1.6	13.05 ± 0.29 ^f^	1.8	5.52 ± 0.09 ^a^	1.8	6.03 ± 0.01 ^a^	1.6	9.10 ± 0.00 ^d^	1.8
Cys	11.12 ± 0.04 ^d^	3.3	16.63 ± 0.01 ^g^	3.3	9.94 ± 0.04 ^c^	1.6	13.62 ± 0.13 ^f^	4.4	19.34 ± 0.52 ^h^	3.8	12.36 ± 0.08 ^e^	1.7	6.73 ± 0.01 ^a^	2.2	11.32 ± 0.04 ^d^	3.1	8.47 ± 0.01 ^b^	1.7
Ile	12.39 ± 0.06 ^b^	3.7	16.78 ± 0.02 ^c^	3.4	36.66 ± 0.25 ^f^	5.8	9.99 ± 0.18 ^a^	3.2	17.40 ± 0.02 ^c^	3.4	31.86 ± 0.72 ^e^	4.5	13.04 ± 0.24 ^b^	4.2	12.57 ± 0.07 ^b^	3.4	22.27 ± 0.22 ^d^	4.5
Leu	17.01 ± 0.17 ^b^	5.1	23.83 ± 0.03 ^d^	4.8	35.06 ± 0.06 ^g^	5.5	15.18 ± 0.02 ^a^	4.9	25.38 ± 0.05 ^e^	5	40.39 ± 0.55 ^h^	5.7	18.89 ± 0.01 ^c^	6.1	18.47 ± 0.05 ^c^	5	29.62 ± 0.18 ^f^	5.9
Phe	12.83 ± 0.08 ^b^	3.9	19.17 ± 0.66 ^c^	3.9	28.83 ± 0.46 ^e^	4.6	9.59 ± 0.03 ^a^	3.1	18.28 ± 0.02 ^c^	3.6	34.63 ± 0.77 ^f^	4.9	14.09 ± 0.01 ^b^	4.5	12.85 ± 0.24 ^b^	3.5	23.76 ± 0.02 ^d^	4.7
Lys	13.56 ± 0.08 ^b^	4.1	20.41 ± 0.25 ^e^	4.1	19.47 ± 0.15 ^d^	3.1	14.32 ± 0.18 ^b^	4.6	20.74 ± 0.03 ^e^	4.1	22.84 ± 0.41 ^f^	3.2	12.06 ± 0.10 ^a^	3.9	16.84 ± 0.23 ^c^	4.6	16.44 ± 0.23 ^c^	3.3
Trp	4.20 ± 0.04 ^b^	1.3	8.80 ± 0.09 ^e^	1.8	11.48 ± 0.13 ^g^	1.8	4.51 ± 0.01 ^b^	1.4	6.75 ± 0.21 ^d^	1.3	10.97 ± 0.04 ^f^	1.5	3.81 ± 0.01 ^a^	1.2	4.66 ± 0.05 ^c^	1.3	6.63 ± 0.04 ^b^	1.3
**Total**	**332.55** ± 2.5 ^a^	100	**496.72** ± 5.18 ^c^	100	**633.02** ± 2.34 ^d^	100	**311.87** ± 1.6 ^a^	100	**505.82** ± 2.14 ^c^	100	**708.14** ± 15.1 ^e^	100	**311.25** ± 1.5 ^a^	100	**369.73** ± 1.88 ^b^	100	**500.43** ± 4.0 ^c^	100
SCAA	5		4.8		3.3		6.1		5.4		3.6		3.9		4.7		3.5	
BCAA	13.4		12		16.2		11.9		12.3		15.5		15.2		12.4		15.7	
HAA	34		31.7		35.9		32.8		31.9		36.3		35.9		32.2		36	
NCAA	37		40.3		37.5		37.7		39.8		36.4		35.5		39.7		36.6	
PCAA	14.3		14.5		13.6		14.7		14.1		14.1		14.4		14.4		14.1	

WEF–water-extracted fraction; SEF–salt-extracted fraction; AEF–alkaline-extracted fraction. Amino acid abbreviation: Asx–aspartic acid + asparagine, Glx–glutamic acid + glutamine, Ser–serine, Gly–glycine, His–histidine, Arg–arginine, Thr–threonine, Ala–alanine, Pro–proline, Tyr–tyrosine, Val–valine, Met–methionine, Cys–cysteine, Ile–isoleucine, Leu–leucine, Phe–phenylalanine, Lys–lysine, Trp–tryptophan. SCAA–sulphur-containing amino acids (Met, Cys; antioxidant activity); BCAA–sum of branched-chain amino acids (Val, Leu, Ile; hypotensive activity); HAA–sum of hydrophobic amino acids (Gly, Ala, Pro, Val, Met, Leu, Ile, Phe, Trp); NCAA–negatively charged amino acids (Asx, Glx; reducing activity); PCAA–positively charged amino acids (His, Lys, Arg). Mean (n = 4) ± SD. In each row (amino acid), means marked with different superscript letters are significantly different (Tukey’s test at α = 0.05).

**Table 2:** SDG and phenolic acid content (mg g^−1^) in the flaxseed protein fractions.

Fraction	Compounds		
	SDG	*p*-Coumaric Acid	Ferulic Acid
Szafir cultivar			
WEF	2.80 ± 0.16 ^b^	0.10 ± 0.004 ^b^	0.51 ± 0.01 ^b^
SEF	6.01 ± 0.40 ^c^	0.20 ± 0.02 ^d^	0.79 ± 0.02 ^c^
AEF	14.26 ± 0.03 ^f^	0.69 ± 0.002 ^g^	2.52 ± 0.01 ^f^
Oliwin cultivar			
WEF	1.72 ± 0.03 ^a^	0.05 ± 0.001 ^a^	0.39 ± 0.002 ^a^
SEF	3.43 ± 0.27 ^b^	0.10 ± 0.01 ^b^	0.52 ± 0.07 ^b^
AEF	9.53 ± 0.46 ^e^	0.45 ± 0.04 ^f^	2.32 ± 0.11 ^e^
Jantarol cultivar			
WEF	5.84 ± 0.29 ^c^	0.10 ± 0.01 ^b^	0.32 ± 0.03 ^a^
SEF	7.73 ± 0.67 ^d^	0.14 ± 0.02 ^c^	0.62 ± 0.02 ^b^
AEF	19.06 ± 0.18 ^g^	0.38 ± 0.01 ^e^	1.54 ± 0.004 ^d^
*p*	<0.001	<0.001	<0.001

WEF—water-extracted fraction; SEF—salt-extracted fraction; AEF—alkaline-extracted fraction; SDG—secoisolariciresinol diglucoside. Mean (n = 4) ± SD. In each column (compound), means marked with different superscript letters are significantly different (*p* value for ANOVA, Tukey’s test, at α = 0.05).

**Table 3:** Functional properties of the flaxseed protein fractions.

Property	Protein Fractions			*p*
/Cultivar	WEF	SEF	AEF	
**Surface Hydrophobicity (H_o_) ^1^**				
Szafir	7.30 ± 0.51 ^a^	16.13 ± 0.43 ^c^	67.33 ± 0.32 ^f^	
Oliwin	6.52 ± 0.22 ^a^	14.49 ± 0.46 ^c^	50.80 ± 0.63 ^e^	
Jantarol	10.30 ± 0.39 ^b^	21.34 ± 0.88 ^d^	80.93 ± 0.94 ^g^	
				<0.001
Antiradical activity (ORAC_FL) ^2^				
Szafir	141 ± 1 ^b^	352 ± 16 ^d^	894 ± 35 ^f^	
Oliwin	131 ± 6 ^ab^	252 ± 11 ^c^	627 ± 15 ^e^	
Jantarol	95 ± 11 ^a^	317 ± 5 ^d^	1356 ± 22 ^g^	
				<0.001
Total phenolic content (TPC)/compounds reducing FCR ^3^				
Szafir	8.95 ± 0.54 ^b^	16.91 ± 0.34 ^e^	32.03 ± 0.95 ^h^	
Oliwin	10.67 ± 0.43 ^c^	15.27 ± 0.84 ^d^	28.44 ± 0.49 ^g^	
Jantarol	6.98 ± 0.25 ^a^	20.76 ± 0.41 ^f^	42.21 ± 1.17 ^i^	
				<0.001

WEF—water-extracted fraction; SEF—salt-extracted fraction; AEF—alkaline-extracted fraction. ^1^ expressed as index of protein surface hydrophobicity Ho (Ho is defined as the initial slop of the plot of the relative fluorescence intensity vs. protein concentration calculated by the simple linear regression); ^2^ expressed as Trolox equivalent (μM g^−1^ fraction); ^3^ expressed as meq. gallic acid (mg g^−1^ fraction). Mean (n = 4) ± SD. For each functional property, means marked with different superscript letters are significantly different (*p* value for ANOVA, Tukey’s test at α = 0.05).

**Table 4:** Protective effect of the flaxseed protein fractions against oxidation in emulsion system.

Cultivar		Antioxidant/Protein Fraction					
		Control	BHT	WEF	SEF	AEF	*p*
			0.1 g L^−1^	10 g L^−1^	10 g L^−1^	10 g L^−1^	
**CD (g kg^−1^)**							
	initial	0.25 ± 0.01	0.24 ± 0.01	0.26 ± 0.02	0.23 ± 0.01	0.25 ± 0.01	0.174
Szafir	after 24 h	1.19 ± 0.12 ^c^	0.54 ± 0.03 ^b^	0.45 ± 0.01 ^b^	0.29 ± 0.02 ^a^	0.44 ± 0.05 ^b^	<0.001
	**PI_24 h**		**2.2**	**2.6**	**4.1**	**2.7**	
	initial	0.33 ± 0.02	0.32 ± 0.01	0.32 ± 0.01	0.31 ± 0.01	0.32 ± 0.01	0.055
Oliwin	after 24 h	0.90 ± 0.01 ^c^	0.51 ± 0.04 ^b^	0.47 ± 0.04 ^b^	0.32 ± 0.03 ^a^	0.53 ± 0.02 ^b^	<0.001
	**PI_24 h**		**1.8**	**1.9**	**2.8**	**1.7**	
	initial	0.26 ± 0.02	0.26 ± 0.02	0.27 ± 0.01	0.24 ± 0.02	0.27 ± 0.01	0.24
Jantarol	after 24 h	1.08 ± 0.13 ^c^	0.53 ± 0.03 ^b^	0.46 ± 0.01 ^b^	0.31 ± 0.02 ^a^	0.52 ± 0.04 ^b^	<0.001
	**PI_24 h**		**2**	**2.3**	**3.5**	**2.1**	
**CT**							
	initial	0.19 ± 0.01 ^a^	0.20 ± 0.01 ^ab^	0.21 ± 0.02 ^b^	0.21 ± 0.01 ^ab^	0.22 ± 0.01 ^b^	0.002
Szafir	after 24 h	0.46 ± 0.05 ^c^	0.28 ± 0.01 ^b^	0.26 ± 0.01 ^ab^	0.22 ± 0.01 ^a^	0.26 ± 0.01 ^ab^	<0.001
	**PI_24 h**		**1.7**	**1.8**	**2.1**	**1.8**	
	initial	0.24 ± 0.01 ^a^	0.26 ± 0.01 ^ab^	0.26 ± 0.02 ^ab^	0.25 ± 0.01 ^a^	0.28 ± 0.01 ^b^	0.006
Oliwin	after 24 h	0.36 ± 0.01 ^c^	0.28 ± 0.01 ^ab^	0.28 ± 0.01 ^ab^	0.26 ± 0.03 ^a^	0.31 ± 0.01 ^b^	<0.001
	**PI_24 h**		**1.3**	**1.3**	**1.4**	**1.2**	
	initial	0.20 ± 0.01 ^a^	0.21 ± 0.01 ^a^	0.21 ± 0.01 ^a^	0.24 ± 0.01 ^b^	0.25 ± 0.01 ^b^	0.006
Jantarol	after 24 h	0.42 ± 0.05 ^c^	0.29 ± 0.01 ^b^	0.26 ± 0.01 ^ab^	0.24 ± 0.01 ^a^	0.30 ± 0.01 ^b^	<0.001
	**PI_24 h**		**1.4**	**1.6**	**1.8**	**1.4**	

Emulsion samples: Control—without any fraction or antioxidant addition, BHT—antioxidant butylated hydroxytoluene, WEF—water-extracted fraction, SEF—salt-extracted fraction, AEF—alkaline-extracted fraction, CD—conjugated dienoic derivatives, CT—conjugated trienoic derivatives; initial—CD/CT values before incubation, after 24 h—CD/CT values after 24 h-incubation at 40 °C; PI_24 h–protective index after 24 h incubation of emulsion at 40 °C (defined as the CD/CT value of the sample with the antioxidant or protein fraction divided by the CD/CT value of the control sample): PI > 1 denotes a protective effect against oxidation in emulsion. Mean (n = 4) ± SD. In each row, means marked with different superscript letters are significantly different (*p* value for ANOVA, Tukey’s test, at α = 0.05).

## Data Availability

The data presented in this study are available in the article and as Supplementary Materials.

## Supplementary Materials

The following supporting information can be downloaded at: https://www.mdpi.com/article/10.3390/antiox12030675/s1. Table S1: Results of statistical analysis for the effect of subsequent fractionations on changing in the percentage share of protein bands in SDS-PAGE electrophoresis.
