# Supplementary material for: Protein Fractions from Flaxseed: The Effect of Subsequent Extractions on Composition and Antioxidant Capacity
Source: Antioxidants (Basel). 2023 Mar 9;12(3):675. doi: 10.3390/antiox12030675 (PMC10045795; doi:10.3390/antiox12030675)
Supplement: Supplementary file 1 [file antioxidants-12-00675-s001.zip › antioxidants-2156078-supplementary.pdf]

Protein Fractions from Flaxseed: The Effect of Subsequent Extractions on Composition and Antioxidant Capacity

Katarzyna Waszkowiak, Beata Mikołajczak, Katarzyna Polanowska, Marek Wieruszewski, Przemysław Siejak, Wojciech Smułek, and Maciej Jarzębski

Supplementary materials

Antioxidants 2023, 11, x. https://doi.org/10.3390/xxxxx

Table S1. Results of statistical analysis for the effect of subsequent fractionations on changing in the percentage share of protein bands in SDS-PAGE electrophoresis

Band no	Range of molecular weight [kDa]	Protein fractions		
		WEF	SEF	AEF
1	56—38	0.00 ^{b} ± 0.00	0.00 ^{b} ± 0.00	2.03 ^{a} ± 0.67
2		0.78 ^{c} ± 0.45	2.57 ^{a} ± 0.65	1.92 ^{b} ± 0.70
3		0.00 ^{b} ± 0.00	0.00 ^{b} ± 0.00	2.15 ^{a} ± 0.46
4	35—16	9.82 ^{b} ± 5.97	8.23 ^{c} ± 3.16	23.86 ^{a} ± 1.65
5		4.33 ^{b} ± 4.05	1.53 ^{c} ± 0.25	9,43 ^{a} ± 1.00
6		10.57 ^{b} ± 4.03	8,57 ^{c} ± 1.18	12.95 ^{a} ± 1.65
7		12.35 ^{b} ± 3.95	15.92 ^{a} ± 1.71	0.00 ^{c} ± 0.00
8		23.23 ^{b} ± 9.56	16.00 ^{c} ± 2.14	39.55 ^{a} ± 1.95
9	< 15	7.90 ^{b} ± 5.07	8,57 ^{a} ± 2.18	0.00 ^{c} ± 0.00
10		21.78 ^{b} ± 7.79	28.42 ^{a} ± 4.04	8.10 ^{c} ± 3.38
11		9.23 ^{b} ± 5.73	10.20 ^{a} ± 1.43	0.00 ^{c} ± 0.00

No 1-11—number of protein bands on SDS-page electrophoretic gels. WEF—water-extracted fraction; SEF—salt-extracted fraction; AEF—alkaline-extracted fraction. Means (n = 9) ± SD. In each row, means marked with different superscript letters are significantly different (two-way ANOVA and post hoc Tukey's test at α = 0.05).
